# PSME2 exacerbates ulcerative colitis by disrupting intestinal barrier function and promoting autophagy-dependent inflammation

**DOI:** 10.1515/biol-2025-1196

**Published:** 2025-10-30

**Authors:** Min Li, Jing Chen, Shimeng Xu, Zhaoxiu Liu, Cuihua Lu, Sijia Ge

**Affiliations:** Affiliated Hospital of Nantong University, Nantong University, Nantong, Jiangsu, 226001, China

**Keywords:** PSME2, ulcerative colitis, autophagy, intestinal barrier function, pro-inflammatory cytokines

## Abstract

The immunoproteasome regulatory component proteasome activator subunit beta (PSME2) plays a crucial role in immune regulation, yet its impact on intestinal barrier integrity in ulcerative colitis (UC) remains unclear. This study aimed to elucidate the involvement of PSME2 in UC pathogenesis. Clinical samples from UC patients and healthy controls were analyzed to assess PSME2 expression. A dextran sulfate sodium-induced colitis mouse model was employed to evaluate disease progression, colon histology, and PSME2 levels. *In vitro*, colonic cells were treated with lipopolysaccharide (LPS) to examine tight junction protein (claudin-1) expression and inflammatory mediators (IL-6, IL-10, TNF-α). Autophagy modulation was investigated using PSME2 silencing and chloroquine (CQ) treatment. PSME2 upregulation in UC and colitis mice correlated with disease severity. *In vitro*, LPS suppressed claudin-1 expression, while PSME2 knockdown restored claudin-1 levels and reduced inflammatory cytokines. PSME2 depletion enhanced autophagy, as indicated by an increased LC3-II/LC3-I ratio, reduced p62, and elevated LC3B puncta. CQ treatment reversed the protective effects of PSME2 silencing, confirming autophagy’s role in barrier integrity. PSME2 exacerbates intestinal inflammation by promoting cytokine release and disrupting epithelial barrier function through autophagy dysregulation. Suggesting its potential as a therapeutic target.

## 1 Introduction

Ulcerative colitis (UC) is a group of chronic, relapsing inflammatory disorders of the gastrointestinal tract with multifactorial etiology [1]. In recent decades, UC incidence has risen worldwide, coinciding with changes in environmental exposures, lifestyle, and gut microbiota composition [2]. The precise etiopathogenesis of UC remains incompletely understood. Current evidence implicates a complex interplay of genetic predisposition, intestinal dysbiosis, environmental factors, and immune dysregulation in the development of UC [3]. Clinically, early-stage UC often presents with non-specific symptoms such as abdominal discomfort, altered bowel habits, hematochezia, fatigue, and iron-deficiency anemia [4]. These vague symptoms can lead to diagnostic delays. As a result, many patients are not diagnosed until they develop advanced complications [5]. This diagnostic challenge underscores the need for novel biomarkers to facilitate earlier detection, guide therapy, and improve prognostic stratification in UC.

Proteasome activator subunit beta (PSME2) is a core regulatory subunit of the immunoproteasome complex and orchestrates critical cellular processes, including protein homeostasis, antigen presentation, and immune signaling by modulating proteasome activity [6]. Emerging evidence from oncology indicates that PSME2 is dysregulated in multiple malignancies and plays diverse pathological roles [7]. For example, PSME2 overexpression can suppress autophagic flux via the mTOR/Beclin-1 pathway to promote renal cell carcinoma metastasis [8]; it has been implicated in transcriptional activation in Burkitt’s lymphoma [8]; and it serves as a diagnostic marker in cutaneous melanoma and a predictor of lymph node metastasis in breast carcinoma [9,10]. In the context of gastrointestinal disease, accumulating data suggest a link between PSME2 and UC. Multi-cohort transcriptomic analyses have consistently shown upregulation of PSME2 in UC patients, with similar trends observed in dextran sulfate sodium (DSS)-induced colitis models and clinical specimens [11]. Despite these observations, the precise mechanistic contribution of PSME2 to UC pathophysiology remains undefined. This gap necessitates a systematic investigation of PSME2’s molecular interactions within the intestinal inflammatory cascade.

To address this, we established a comprehensive experimental framework incorporating both *in vivo* and *in vitro* systems to characterize PSME2’s expression and function in UC. Using multiple validation methods (quantitative real-time RT-PCR [qRT-PCR], western blotting, and immunohistochemistry [IHC]), we confirmed that PSME2 is dysregulated in UC models. Functional studies with genetic knockdown of PSME2 demonstrated that PSME2 inhibition attenuates lipopolysaccharide (LPS)-induced inflammatory responses via enhancement of autophagy in intestinal epithelial cells. These findings not only provide potential molecular markers for early, non-invasive UC diagnosis but also highlight a novel therapeutic strategy: targeting PSME2 to modulate autophagy and inflammatory pathways in UC.

## 2 Materials and methods

### 2.1 Clinical specimens

Colon tissue specimens were obtained from 40 UC patients (18–80 years of age) at the Affiliated Hospital of Nantong University between August 2023 and June 2024. Diagnoses were confirmed through clinical evaluation, endoscopy, and histopathology. Exclusion criteria for patients included: (1) concurrent intestinal pathologies (such as infectious or radiation enteritis, short bowel syndrome), (2) prior intestinal surgery, and (3) recent treatment with glucocorticoids or biologics. Twenty colonic tissue samples from patients undergoing endoscopic polyp resection (with no inflammatory disease) served as healthy controls. Demographic data (age, sex), laboratory parameters, and endoscopic findings were recorded for all subjects.


**Informed consent:** Informed consent has been obtained from all individuals included in this study.
**Ethical approval:** The research related to human use has been complied with all the relevant national regulations, institutional policies and in accordance with the tenets of the Helsinki Declaration, and has been approved by the Medical Ethics Committee of the Affiliated Hospital of Nantong University Medical School (Approval No: 2020-L092).

### 2.2 Bioinformatics analysis

Gene expression profiles were retrieved from the Gene Expression Omnibus (GEO) repository for comparative transcriptomic analysis. The data included 23 inflamed (lesional) colonic tissues from UC patients and 23 adjacent non-lesional colonic tissues from UC patients (from GSE59071). We compared PSME2 expression between lesional and non-lesional tissues in UC, and also between UC patient samples and normal controls, to identify differential expression patterns of PSME2.

### 2.3 Cell culture and transfection

The normal intestinal epithelium cell line NCM460 and colorectal cancer cell line HT-29 were purchased from American Type Culture Collection (Rockville, MD, USA).

Cells were cultured in DMEM containing 10% fetal bovine serum and 1% penicillin–streptomycin, under standard conditions of 37°C with 5% CO_2_. When they reached approximately 70% confluence in six-well plates, the cells were prepared for transfection. PSME2 was silenced using a specific small interfering RNA. A scrambled siRNA served as a negative control. Transfections were performed with Lipofectamine 3000 (Thermo Fisher) according to the manufacturer’s instructions. The siRNA sequences were si-NC: sense 5′-UUCUCCGAACGUGUCACGUTT-3′, antisense 5′-ACGUGACACGUUCGGAGAATT-3′; si-PSME2#2: sense 5′-CAGAGAUCUAGCGACUGAAGC-3′, antisense 5′-UUCAGUCGCUAGAUCUCUGGU-3′ (Shanghai GenePharma Co., Ltd).

### 2.4 Animal experiments

From the Animal Center of Nantong University Medical School, we obtained 6–8-week-old male C57BL/6 mice, which were housed under SPF conditions and provided with food and water freely. At 8–10 weeks of age, mice were randomly assigned to experimental groups. Each experimental group consisted of six male C57BL/6 mice, with six mice in the DSS treatment group and six mice in the control group.

For induction of colitis, mice received 2.5% DSS (MW ∼40 kDa; MP Biomedicals) in their drinking water *ad libitum* for 7 days [12]. Fresh DSS solution was provided every 48 h. Throughout the experiment, daily assessments of body weight and disease activity index (DAI) were performed for all 12 mice. No mortality was observed in any of the 12 mice during the study. The following criteria are used: weight loss between 1 and 5% is assigned 1 point, 5–10% is 2 points, 10–15% is 3 points, and over 15% is 4 points. For rectal fecal occult blood, weak positive results are given 1 point, positive results 2 points, visible blood in the stool 3 points, and rectal bleeding without defecation 4 points. Stool consistency is graded as follows: slightly loose stools are 1 point, soft but formed stools are 2 points, very loose stools are 3 points, and diarrhea or watery stools are 4 points. No weight loss, negative occult blood, and normal stool consistency score 0 points. The total DAI score ranges from 0 to 12 points [13].

On Day 8, mice were euthanized. Colons were removed and measured for length. Distal colon segments were fixed in 4% paraformaldehyde (24 h) for paraffin embedding and subsequent histological and immunostaining analyses. Remaining colon tissue was snap-frozen in liquid nitrogen for molecular analyses.


**Ethical approval:** The research related to animal use has been complied with all the relevant national regulations and institutional policies for the care and use of animals, and has been approved by the Animal Ethics Committee of Nantong University Medical School (Approval No: S20200315-009).

### 2.5 Western blotting

Total proteins were isolated from human colonic tissues (including samples from UC patients and healthy controls), mouse colons (with or without DSS treatment), as well as cultured cell lysates. For each sample, equal amounts of protein were subjected to SDS-PAGE, followed by transfer onto PVDF membranes. The membranes were first blocked in TBST buffer containing 5% skim milk for 2 h at room temperature and then incubated overnight at 4°C with primary antibodies. The following antibodies were used: PSME2 (1:1,000; Proteintech), claudin-1 (1:1,000; Abcam), LC3-I/II (1:500; Novus Biologicals), p62/SQSTM1 (1:500; Cell Signaling Technology), β-tubulin (1:5,000; Proteintech), and GAPDH (1:5,000; Proteintech). After thorough washing, HRP-conjugated secondary antibodies (1:5,000; Jackson ImmunoResearch) were applied for 2 h at room temperature. Protein bands were visualized using an ECL chemiluminescence substrate (NCM Biotech) and imaged with the ChemiDoc system. Densitometric analysis was performed with ImageJ (NIH), and relative expression was normalized to internal loading controls.

### 2.6 Cell viability assay

Cell viability was determined using the Cell Counting Kit-8 (CCK-8; Solarbio, China). NCM460 and HT29 cells were seeded into 96-well plates at a density of 5 × 10³ cells/well after transfection and then exposed to graded concentrations of LPS (0–10 μg/mL for NCM460 and 0–100 μg/mL for HT29). After incubation, 10 μL of CCK-8 reagent was added to each well, followed by 1–2 h incubation at 37°C in 5% CO_2_ under dark conditions. Absorbance was measured at 490 nm using a spectrophotometer (Bio-Rad, USA). The viability of treated cells was expressed as a percentage relative to the control group (set as 100%).

### 2.7 qRT-PCR

Total RNA was extracted from cultured cell samples using TRIzol^®^ reagent (Invitrogen). RNA was reverse-transcribed into cDNA using a High-Capacity cDNA kit (Applied Biosystems). Quantitative PCR was performed using SYBR Green Master Mix (Thermo Fisher) on an ABI StepOnePlus thermocycler. Gene-specific primers were as follows: PSME2 forward 5′-CTTTTTCCAGGAGGCTGAGGAAT-3′, reverse 5′-AGGGAAGTCAAGTCAGCCAC-3′; IL-6 forward 5′-ACTTCCATCCAGTTGCCTTCTTGG-3′, reverse 5′-TTAAGCCTCCGACTTGTGAAGTGG-3′; TNF-α forward 5′-AAGCCTGTAGCCCACGTCGTA-3′, reverse 5′-GGCACCACTAGTTGGTTGTCTTTG-3′; IL-10 forward 5′-CTGCTATGCTGCCTGCTCTTACTG-3′, reverse 5′-ATGTGGGCTCTGGCCGACTGG-3′; GAPDH (housekeeping control) forward 5′-CAGGAGGCATTGCTGATGAT-3′, reverse 5′-GAAGGCTGGGGGCTCATTT-3′. Relative mRNA expression was calculated by the 2^(−ΔΔCt)^ method, using GAPDH for normalization.

### 2.8 Histopathological analysis

Paraffin-embedded colon tissues were sectioned at 4 μm and stained with hematoxylin and eosin (H&E). Histopathological changes were evaluated under light microscopy. Two independent, blinded investigators scored the colonic inflammation and tissue damage using established criteria (assessing features such as inflammatory cell infiltration, crypt architecture distortion, goblet cell loss, and ulceration). Scores from the two evaluators were averaged for each sample.

The H&E staining was evaluated based on the following criteria: inflammation: a score of 0 indicates no inflammation, while mild, moderate, and severe inflammation are scored as 1, 2, and 3, respectively. Lesion depth: a score of 0 corresponds to the absence of lesions. Lesions confined to the mucosa are scored as 1, extending to the submucosa as 2, and reaching the serosal layer as 3. Crypt destruction: no destruction is scored as 0, basal crypt destruction up to 1/3 is scored as 1, 2/3 destruction as 2, retention of only surface epithelium as 3, and complete destruction of crypts and epithelium as 4. Mucosal regeneration: normal mucosal tissue is scored as 0, nearly complete regeneration as 1, partial regeneration with only epithelial repair as 2, epithelial damage as 3, and no repair as 4. Lesion extent: a lesion involving 1–25% of the area is scored as 1, 26–50% as 2, 51–75% as 3, and 76–100% as 4. The final score is the cumulative total of these five parameters, with a range of 0–18 points [14].

### 2.9 IHC

Paraffin-embedded and formalin-fixed colon tissues from both human and mouse samples were prepared for IHC. After deparaffinization and rehydration, antigen retrieval was carried out using citrate buffer (pH 6.0) at 95°C for 15 min. Endogenous peroxidase activity was quenched by incubation with 3% H₂O₂, followed by blocking with 5% bovine serum albumin (BSA) for 1 h. The tissue sections were then exposed to the primary antibody against PSME2 (1:200) and incubated overnight at 4°C. After washing, appropriate HRP-conjugated secondary antibodies were applied for 30 min at room temperature. Visualization of immunoreactivity was achieved with DAB substrate, and the nuclei were counterstained with hematoxylin. Images were acquired using a bright-field microscope, and positive signals (brown DAB staining) were semi-quantitatively assessed based on their intensity and distribution within the tissues.

For quantitative analysis, first, digital image analysis was performed using ImageJ software. The integrated optical density (IOD) of PSME2-positive staining was measured, and the average optical density (OD = IOD/area) was calculated to reflect relative expression levels.

### 2.10 Immunofluorescence (IF)

Cultured cells (NCM460 or HT29) grown on glass coverslips were used for IF staining. After treatments, cells were washed with PBS and fixed in 4% paraformaldehyde for 20 min at room temperature. Cells were washed again, then permeabilized with 0.2% Triton X-100 for 15 min. After additional washes, nonspecific binding was blocked by incubating cells in 5% BSA for 1 h. Cells were then incubated overnight at 4°C with primary antibodies (for example, anti-LC3B antibody for detecting autophagosomes). The next day, slides were washed and incubated with fluorophore-conjugated secondary antibodies (e.g., Alexa Fluor 594) for 1 h at room temperature in the dark. Nuclei were counterstained with DAPI for 5 min. Finally, coverslips were mounted on slides with anti-fade mounting medium. Fluorescent images were captured using a fluorescence microscope, and punctate LC3 staining (autophagosome formation) was quantified by counting puncta per cell in multiple fields.

### 2.11 Statistical analysis

Data were analyzed using GraphPad Prism 10 (GraphPad Software) and SPSS 27.0 (IBM). All experiments were performed at least in triplicate. Results are presented as mean ± standard error of the mean (SEM). For comparisons between two groups, Student’s *t*-test was used (unpaired, two-tailed). For multi-group comparisons, one-way ANOVA followed by appropriate *post-hoc* tests was employed. Categorical data were compared using the *χ*² test. A *p*-value < 0.05 was considered statistically significant. Significance levels are indicated as *p < 0.05, **p < 0.01, ***p < 0.001, and ****p < 0.0000.

## 3 Results

### 3.1 Increased PSME2 expression in colonic tissues of UC patients

**Figure 1:**
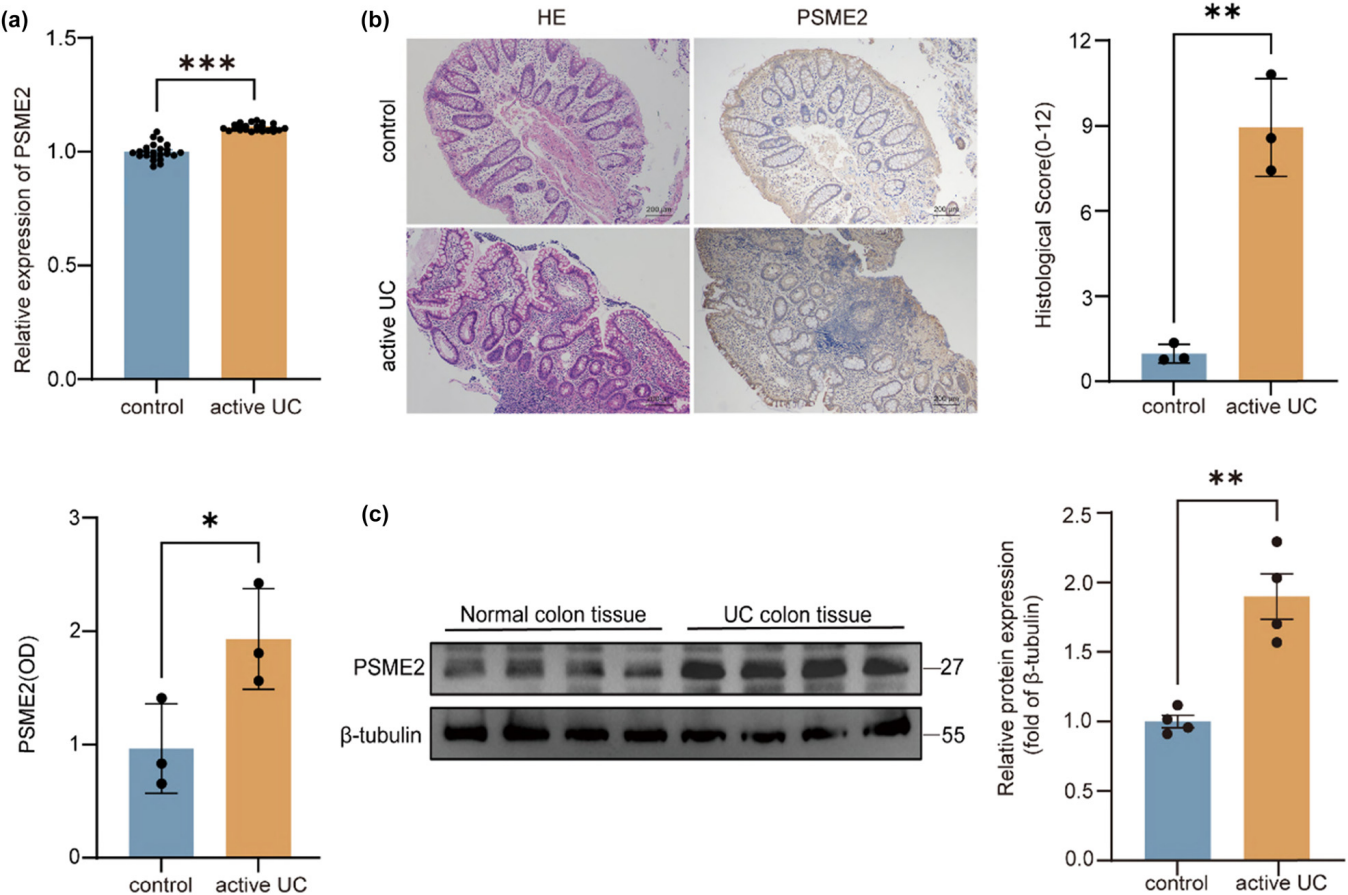
Increased PSME2 expression in diseased colonic tissues of patients with UC. (a) Differential expression of PSME2 between normal human colon tissues (*n* = 23) and diseased colon tissues (*n* = 23) from unpaired samples of UC patients. (b) H&E staining of colonic samples from normal individuals and UC patients; IHC staining to detect the expression level of PSME2 in the colons of normal (*n* = 20) and UC patients (*n* = 40). Quantification of histological scores and PSME2 OD values is shown. Scale bars: 200 μm. (c) Western blot analysis of PSME2 protein levels in colonic tissues from normal controls (*n* = 20) and UC patients (*n* = 40), with β-tubulin as the loading control. Quantification of relative protein expression is shown. Data are presented as mean ± SEM. *p < 0.05, **p < 0.01, ***p < 0.001.

To explore PSME2’s role in UC pathogenesis, we first examined its expression in human colonic tissues. Transcriptomic analysis of public GEO datasets revealed significantly higher PSME2 mRNA levels in inflamed colonic mucosa from UC patients compared to normal controls (Figure 1a). We validated this finding in our clinical samples: PSME2 expression was assessed in colon tissues from 40 UC patients and 20 healthy controls. IHC demonstrated markedly enhanced PSME2 staining in inflamed UC mucosa (Figure 1b). These observations were in line with the characteristic histopathological changes seen in UC tissues: H&E staining showed dense inflammatory cell infiltration in the lamina propria, crypt abscesses, and architectural distortion in UC samples, accompanied by significantly increased histological scores and OD values compared to control tissues (Figure 1b). Consistently, western blot analysis showed that PSME2 protein expression was significantly higher in UC colonic tissues than in controls (Figure 1c). Multivariate analysis of the clinical-pathological correlations revealed that elevated PSME2 expression was independently associated with DAIs while showing no significant correlation with baseline demographic characteristics (age, gender) or subjective symptom severity (abdominal pain score, bloody stool frequency) (Table 1). Collectively, the transcriptomic, histological, and protein expression data indicate that PSME2 is overexpressed in the inflamed intestinal mucosa of UC patients. This upregulation suggests PSME2 may be involved in UC pathogenesis, establishing a foundation for further mechanistic studies.

**Table 1:** Correlation analysis between the expression level of PSME2 and clinicopathologic parameters in 40 patients with UC

Clinicopathological features	PSME2 IHC level		Total	*P* value
	Low (*n* = 14)	High (*n* = 26)		
**Gender**				**0.257**
Male	6 (15.0%)	16 (40.0%)	22	
Female	8 (20.0%)	10 (25.0%)	18	
**Age (years)**				**0.144**
≤37	7 (17.5%)	19 (47.5%)	26	
>37	7 (17.5%)	7 (17.5%)	14	
**Abdominal pain level**				**0.089**
Mild	2 (5.0%)	12 (30.0%)	14	
Moderate	3 (7.5%)	6 (15%)	9	
Severe	9 (22.5%)	8 (20.0%)	17	
**Fecal occult blood**				**0.185**
Negative	9 (22.5%)	17 (42.5%)	26	
Positive	5 (12.5%)	9 (22.5%)	14	
**Disease activity period**				**<0.01**
Remission period	12 (30.0%)	6 (15%)	18	
Active period	2 (5.0%)	20 (50.0%)	22	

### 3.2 PSME2 upregulation in DSS-induced murine colitis

**Figure 2:**
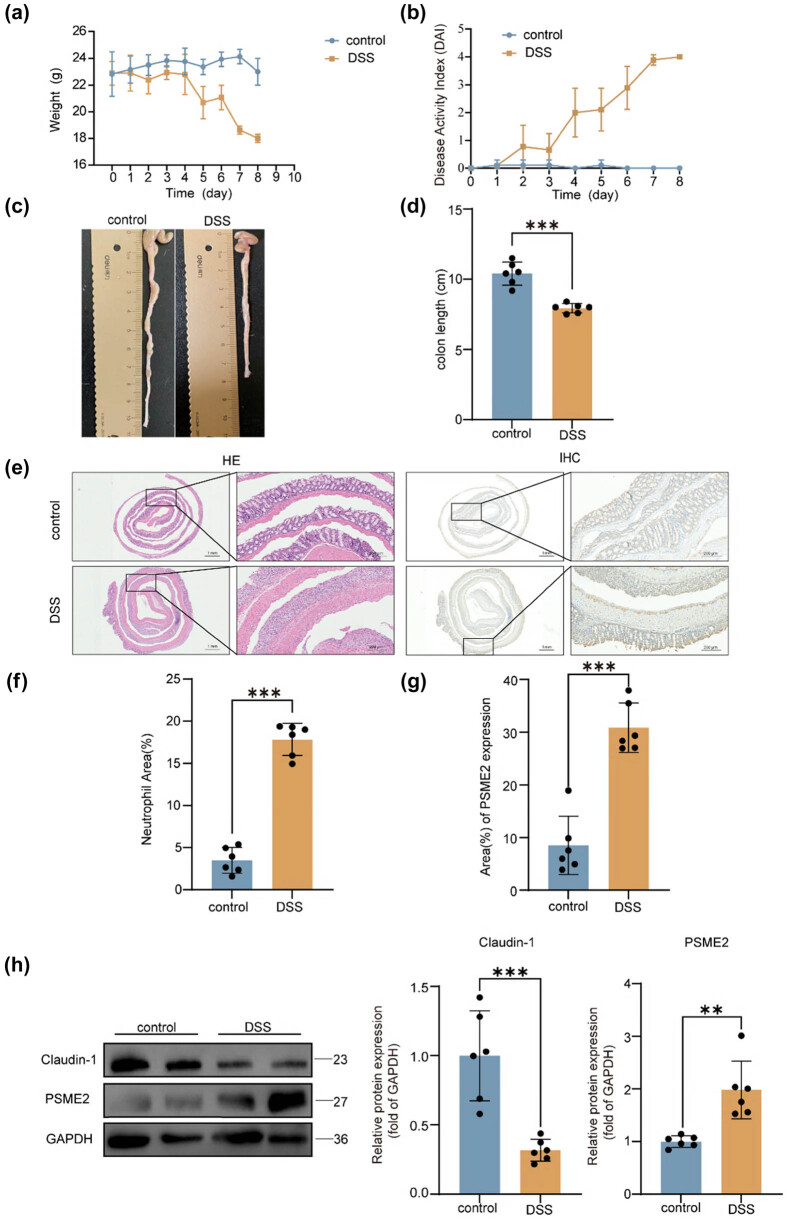
PSME2 expression in the colons of DSS-induced colitis mice. (a) Body weight changes of control and DSS-treated mice (*n* = 6 per group) during the 10-day experiment. (b) DAI scores for control and DSS-treated mice over time (*n* = 6 per group). (c) Representative images of colon tissues from control and DSS-treated mice. (d) Colon length (cm) was significantly shorter in DSS-treated mice compared to controls (*n* = 6 per group). (e) Representative H&E staining and PSME2 IHC images of colonic tissues from control and DSS-treated mice. Scale bars: 100 μm. (f) Quantification of neutrophil area (%) in the colon tissues of control and DSS-treated mice (*n* = 6 per group). (g) Quantification of PSME2 expression area (%) in the colon tissues of control and DSS-treated mice (*n* = 6 per group). (h) Western blot analysis of Claudin-1 and PSME2 protein expression in colon tissues from control and DSS-treated mice. Relative protein expression was quantified and normalized to GAPDH (*n* = 6 per group). Data are presented as mean ± SEM. *p < 0.05, **p < 0.01, ***p < 0.001.

We next investigated PSME2 expression in an *in vivo* model of UC. DSS-induced colitis model was used to mimic intestinal inflammation in mice. Over 7 days of 2.5% DSS administration, mice developed progressive colitis symptoms: body weight declined steadily (Figure 2a), DAI scores rose (Figure 2b), and colon length was significantly reduced compared to untreated controls (Figure 2c). These clinical indicators confirmed successful induction of colitis. Histopathological examination confirmed severe mucosal damage in DSS-treated mice, including epithelial erosion, crypt destruction, and extensive neutrophil infiltration, as revealed by H&E staining (Figure 2e and f). IHC further showed markedly increased PSME2 expression in colonic epithelial cells of DSS-treated mice relative to controls (Figure 2e–g). In parallel, western blotting of colon tissue lysates demonstrated that DSS colitis caused a significant increase in PSME2 protein, accompanied by a decrease in the tight junction protein claudin-1 (Figure 2h). The loss of claudin-1 is consistent with impaired barrier integrity during colitis. Importantly, the pattern of PSME2 upregulation in DSS-treated mice mirrors that observed in UC patient tissues. This concordance strengthens the relevance of the animal model and implicates PSME2 in the disruption of intestinal barrier function during inflammatory disease progression.

### 3.3 PSME2 upregulation in LPS-stimulated colonic cell models

**Figure 3:**
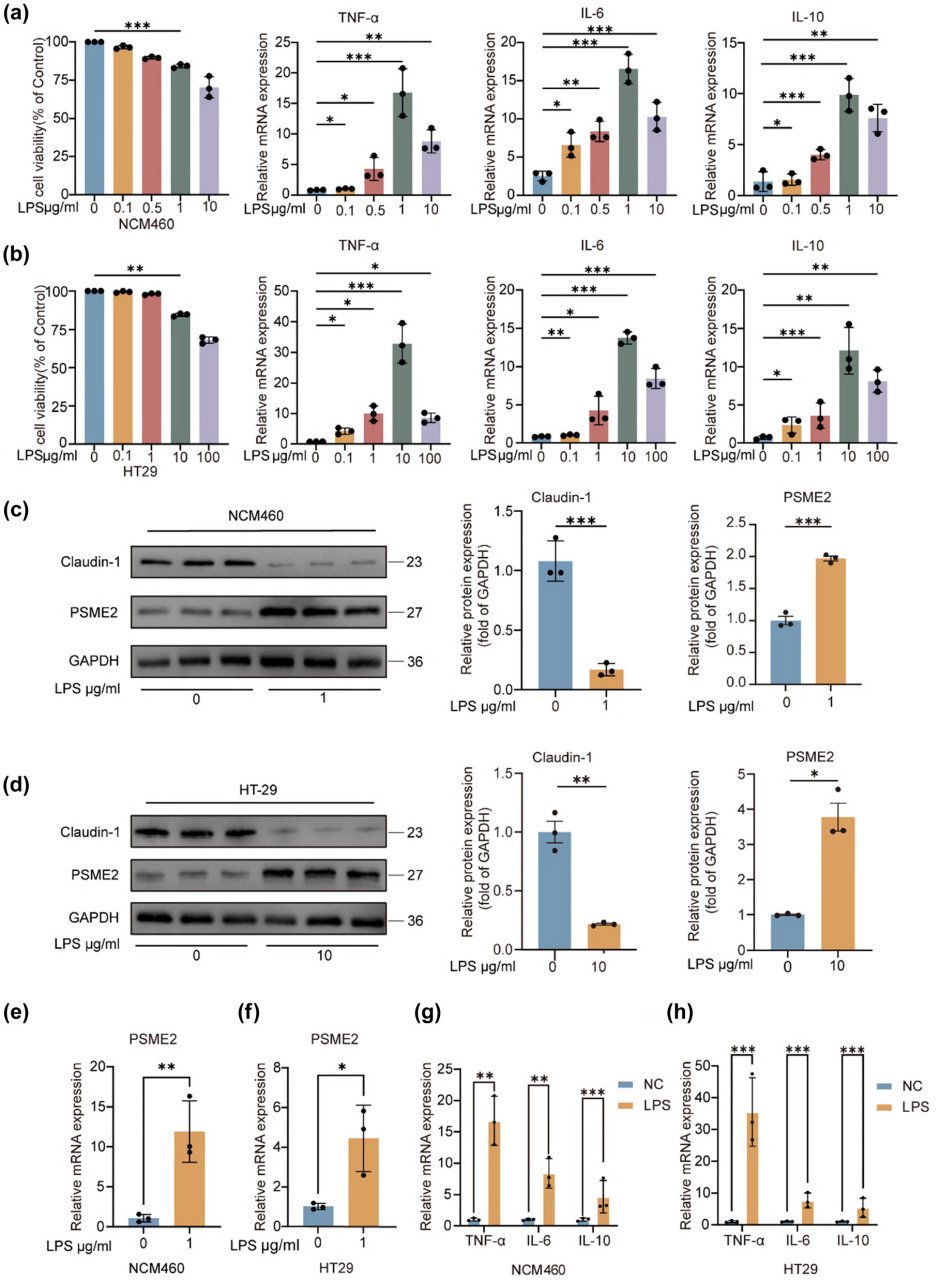
LPS induces upregulated PSME2 expression in an *in vitro* inflammation model. (a) and (b) Cell viability and mRNA expression of TNF-α, IL-6, and IL-10 were assessed in NCM460 and HT29 cells following LPS stimulation at the indicated concentrations for 24 h (*n* = 3 independent biological replicates). (c) and (d) Representative western blot images and quantification of Claudin-1 and PSME2 protein expression in NCM460 and HT29 cells treated with LPS (*n* = 3). GAPDH was used as the loading control. (e) and (f) qRT-PCR analysis of PSME2 mRNA expression in NCM460 and HT29 cells with or without LPS treatment (*n* = 3). (g and h) Relative mRNA expression of TNF-α, IL-6, and IL-10 in NCM460 and HT29 cells with or without LPS stimulation (*n* = 3). Data are presented as mean ± SEM. *p < 0.05, **p < 0.01, ***p < 0.001.

To investigate the role of PSME2 in intestinal inflammation, we established an *in vitro* colonic cell model using LPS stimulation. Dose–response experiments showed that cell viability decreased progressively with increasing LPS concentrations in both NCM460 and HT29 cells. Interestingly, the expression patterns of inflammatory cytokines differed between the two cell lines: in NCM460 cells, TNF-α, IL-6, and IL-10 mRNA levels peaked at 1 μg/mL LPS, whereas in HT29 cells, maximal induction occurred at 10 μg/mL LPS. Notably, at these optimal concentrations, cell viability was only minimally reduced (Figure 3a and b), indicating that the chosen conditions effectively triggered inflammatory responses without causing excessive cytotoxicity. Based on these results, 1 μg/mL LPS was selected for NCM460 cells, while 10 μg/mL LPS was used for HT29 cells to mimic inflammatory insult.

LPS exposure significantly decreased claudin-1 protein levels in both NCM460 and HT29 cells (Figure 3c and d), indicating compromised epithelial barrier protein expression. Concurrently, LPS treatment led to increased PSME2 protein expression in these cells (Figure 3c and d). At the mRNA level, qRT-PCR showed that 24 h of LPS stimulation induced higher expression of PSME2 as well as inflammatory cytokines IL-6, IL-10, and TNF-α (Figure 3e and h). Together, these findings demonstrate that LPS stimulation induces PSME2 upregulation in colonic cell models with cell line-specific sensitivity, and that this response is associated with reduced barrier protein expression and enhanced inflammatory mediator production.

### 3.4 PSME2 knockdown restores epithelial barrier integrity and attenuates LPS-induced inflammation

**Figure 4:**
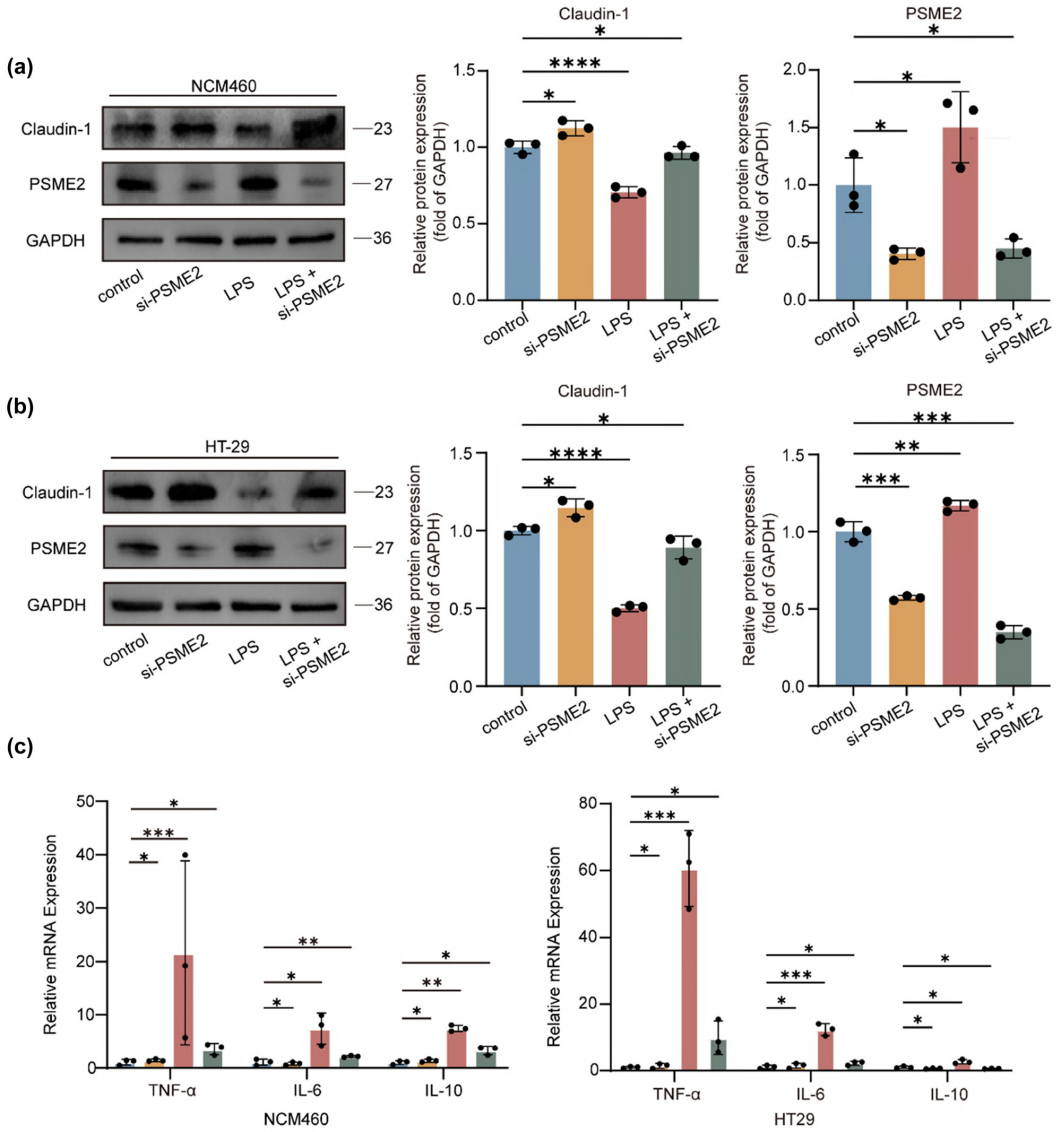
Knockdown of PSME2 ameliorates inflammatory injury in an *in vitro* inflammation model. (a) and (b) Western blotting confirmed the successful knockdown of PSME2 and examined its effect on claudin-1 expression. The control group corresponds to cells transfected with a non-targeting siRNA (siNC), ensuring specificity of the observed effects. Additionally, quantitative analysis was conducted, and the data were presented as mean ± SEM of three independent replicates. (c) qRT-PCR analysis of TNF-α, IL-6, and IL-10 mRNA expression in NCM460 and HT29 cells following LPS stimulation with or without PSME2 knockdown (*n* = 3). Data are presented as mean ± SEM. *p < 0.05, **p < 0.01, ***p < 0.001, ****p < 0.0001.

We next examined whether PSME2 is functionally involved in mediating epithelial barrier damage and inflammatory signaling. PSME2 was silenced in NCM460 and HT29 cells using siRNA, and knockdown efficiency was confirmed by western blotting and qRT-PCR, which showed strong reductions in PSME2 protein and mRNA in siPSME2-transfected cells compared with controls (Figure 4a and b). In NCM460 cells, LPS stimulation markedly decreased claudin-1 expression, whereas PSME2 knockdown significantly preserved claudin-1 protein levels, indicating protection of barrier integrity (Figure 4a). In HT29 cells, claudin-1 was also reduced after LPS exposure (Figure 4b).

Regarding inflammatory signaling, NCM460 cells with PSME2 knockdown exhibited strongly reduced induction of TNF-α and IL-6 in response to LPS, accompanied by lower IL-10 expression compared with control cells (Figure 4c). In HT29 cells, a similar pattern was observed (Figure 4c). Together, these findings indicate that PSME2 facilitates inflammatory epithelial injury by downregulating claudin-1 and promoting cytokine production. Notably, silencing PSME2 exerted a protective effect, particularly in normal epithelial NCM460 cells, by preserving barrier protein expression and attenuating inflammatory responses. Consistent results were also observed in HT29 cells, indicating that this protective mechanism is not restricted to normal epithelial contexts.

### 3.5 PSME2 deficiency enhances autophagic flux in colonic cell models

Our data so far suggested that PSME2 exacerbates inflammation and barrier loss, but the molecular mechanisms required further clarification. Autophagy is a cellular process known to have context-dependent effects on intestinal homeostasis, influencing both barrier integrity and inflammation [15]. We hypothesized that PSME2 might interact with autophagy pathways in epithelial cells. In quiescent or nutrient-deprived states, basal autophagy is generally cytoprotective in the gut: it removes damaged organelles and can improve barrier function by degrading certain pore-forming tight junction proteins [16]. On the other hand, excessive or dysregulated autophagy can be harmful, leading to cell stress or death [17]. Thus, a delicate balance of autophagy is crucial for intestinal epithelial health.

**Figure 5:**
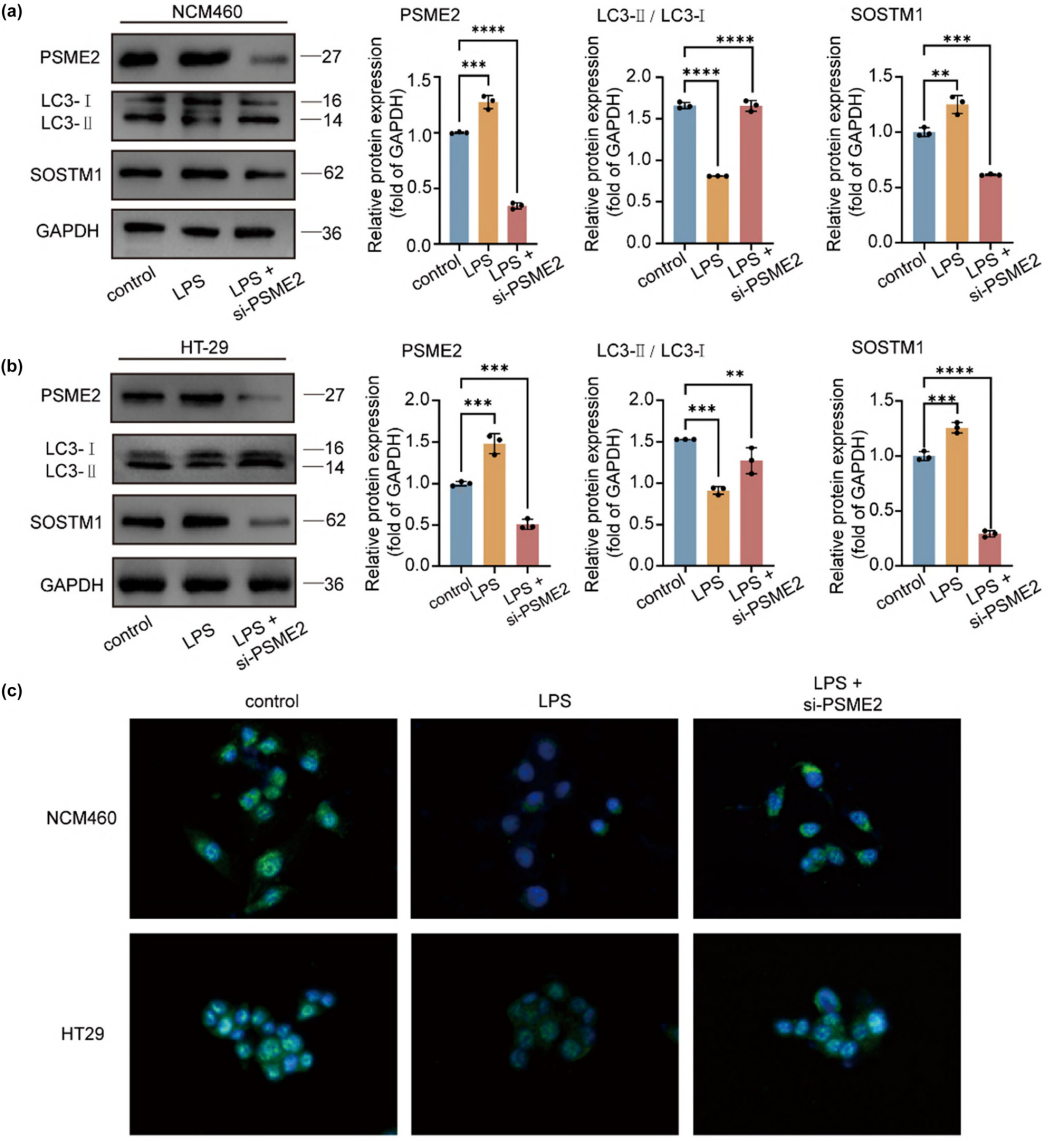
Effect of PSME2 on the autophagic pathway *in vitro*. (a) and (b) Western blot analysis of PSME2, LC3-I/II, and SQSTM1 protein levels in NCM460 and HT29 cells under control, LPS, or LPS + siPSME2 conditions. GAPDH served as the loading control. Protein quantification was performed relative to GAPDH (*n* = 3 independent biological replicates). (c) Representative IF images of LC3B (green) puncta in NCM460 and HT29 cells under the indicated treatments. Nuclei were counterstained with DAPI (blue). Scale bars = 20 μm. Data are presented as mean ± SEM. *p < 0.05, **p < 0.01, ***p < 0.001, ****p < 0.0001.

Since autophagy plays context-dependent roles in intestinal homeostasis, we asked whether PSME2 regulates autophagy under inflammatory conditions. In NCM460 cells, LPS stimulation increased PSME2 expression and was associated with reduced autophagic activity. Silencing PSME2 markedly increased the LC3-II/LC3-I ratio (Figure 5a), accompanied by a strong decrease in p62/SQSTM1 protein levels, indicating enhanced autophagic flux. IF staining further showed that PSME2 knockdown in NCM460 cells led to a robust increase in LC3-II puncta (Figure 5c), confirming autophagosome accumulation. In HT29 cells, a similar pattern was observed: PSME2 knockdown after LPS treatment still elevated the LC3-II/LC3-I ratio and reduced p62 expression (Figure 5b). IF revealed a moderate increase in LC3B puncta in PSME2-deficient HT29 cells (Figure 5c).

Together, these findings indicate that PSME2 deficiency promotes autophagic flux in colonic cell models. Thus, PSME2 appears to function as a negative regulator of autophagy, and its loss enhances autophagic activity under inflammatory stress.

### 3.6 Autophagy inhibition abrogates the protective effects of PSME2 knockdown

**Figure 6:**
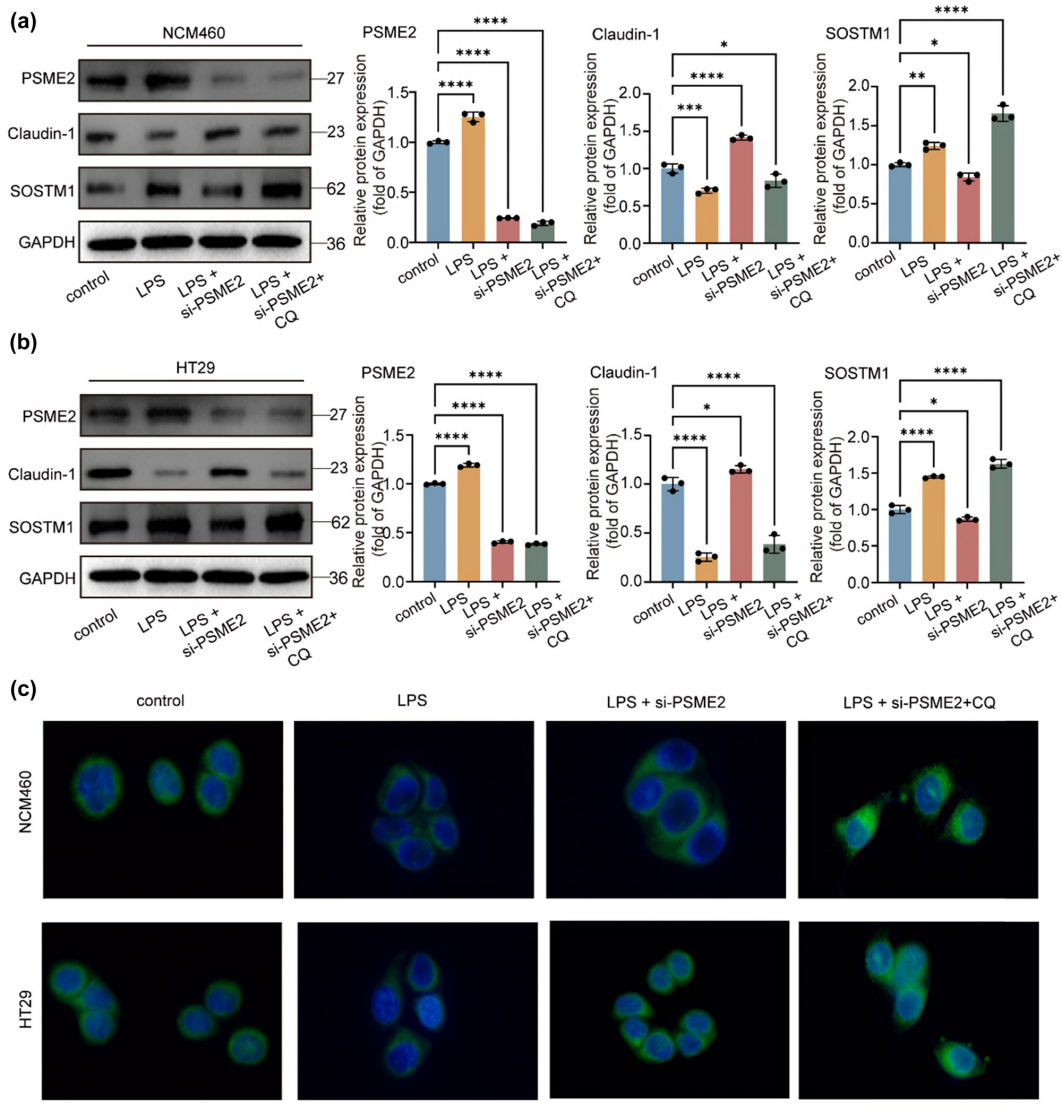
Autophagy inhibition abrogates the protective effects of PSME2 knockdown. NCM 460/HT 29 cells were treated with LPS, siPSME2, and/or CQ (4 μM). (a) and (b) Western blot analysis of PSME2, claudin-1, and SQSTM1/p62 expression in NCM460 (a) and HT29 (b) cells. Compared with the LPS + siPSME2 group, the LPS + siPSME2 + CQ group showed a significant increase in p62 levels but a reduction in claudin-1, indicating that autophagy inhibition reversed the protective effects of PSME2 knockdown. Protein levels were quantified relative to GAPDH (*n* = 3 independent biological replicates). (c) Representative IF images of LC3B (green) puncta in NCM460 and HT29 cells under the indicated conditions. Nuclei were counterstained with DAPI (blue). Scale bars = 40 μm. Data are presented as mean ± SEM. *p < 0.05, **p < 0.01, ***p < 0.001, ****p < 0.0001.

To mechanistically assess whether PSME2-mediated protection against inflammatory damage occurs via autophagy regulation, we employed pharmacological autophagy inhibition with chloroquine (CQ). In NCM460 cells, PSME2 knockdown under LPS challenge significantly upregulated claudin-1 and enhanced autophagic flux, as shown by decreased p62 and increased LC3B puncta; however, CQ treatment reversed these effects, leading to restored p62 accumulation and suppression of claudin-1 expression (Figure 6a and c). In HT29 cells, a similar pronounced trend was observed: CQ treatment in the LPS + siPSME2 group also increased p62 levels and reduced claudin-1 (Figure 6b and c). These definitive findings establish that PSME2 deficiency confers cytoprotection in the intestinal epithelium primarily through autophagy activation, providing novel mechanistic insights into UC pathophysiology.

## 4 Discussion

UC is characterized by chronic relapsing inflammation of the gut, driven by complex interactions among environmental triggers, microbial flora, and host genetic and immune factors [18]. Two fundamental pathological processes underlie UC progression: dysregulation of mucosal immune responses and breakdown of the intestinal epithelial barrier [19]. Although advances in biologics and small-molecule therapies have improved outcomes, a significant proportion of patients exhibit incomplete responses or develop secondary loss of efficacy [11]. This highlights the need to further elucidate novel molecular drivers of UC pathogenesis and to identify therapeutic nodes that may complement existing immunomodulatory strategies. In this context, this study identifies PSME2 as a previously underappreciated regulator of epithelial barrier function and inflammatory signaling, with a particular emphasis on its role in autophagy.

PSME2, a pivotal regulatory component of the immunoproteasome complex, has been implicated in oncogenic processes through its modulation of autophagy pathways [20]. Previous investigations have established that PSME2 overexpression facilitates renal cell carcinoma progression via BNIP3-mediated autophagic regulation [8]. While preliminary clinical observations have suggested a correlation between PSME2 expression levels and intestinal inflammation severity [11], the mechanistic basis for this association has not been systematically investigated. These findings align with the central concept that UC progression involves both epithelial barrier breakdown and immune dysregulation, positioning PSME2 as a dual mediator bridging these processes.

Our data demonstrated that PSME2 is consistently upregulated in UC patients, DSS-induced murine colitis, and LPS-challenged intestinal epithelial cells, suggesting a conserved role in intestinal inflammation. Importantly, PSME2 expression positively correlated with histopathological severity and DAIs, implying potential utility as a biomarker for disease monitoring. Functionally, silencing PSME2 restored expression of the tight junction protein claudin-1 and suppressed pro-inflammatory cytokines, including IL-6 and TNF-α. These findings align with the central concept that UC progression involves both epithelial barrier breakdown and immune dysregulation, positioning PSME2 as a dual mediator bridging these processes.

It is important to note that the two cell models used in this study represent distinct biological contexts. NCM460 cells, derived from normal human colonic epithelium, are suitable for reflecting the role of PSME2 in epithelial homeostasis and barrier function under physiological conditions. In contrast, HT-29 cells, originating from colorectal cancer, exhibit higher basal autophagy and altered claudin-1 expression compared with normal cells, making them more appropriate for mechanistic exploration under tumor-associated pathological conditions. Therefore, conclusions regarding “normal intestinal epithelium” are primarily based on NCM460 and animal data, whereas findings from HT-29 are interpreted as complementary mechanistic insights rather than directly extrapolated to normal physiology.

Autophagy represents an evolutionarily conserved cellular process wherein cytoplasmic components are sequestered within autophagosomes for subsequent lysosomal degradation, serving as a critical mechanism for maintaining metabolic equilibrium and cellular homeostasis [21]. Within the context of inflammatory bowel disease pathogenesis, autophagy contributes to several fundamental biological processes, including adaptive immune responses, lysosomal degradation pathways, and immune-mediated clearance mechanisms [22]. The functional role of autophagy in UC has been increasingly recognized as exhibiting a biphasic nature. Under physiological conditions, basal autophagic activity exerts cytoprotective effects through multiple mechanisms: selective removal of damaged organelles, maintenance of intestinal epithelial cell turnover and homeostasis, and enhancement of barrier function through regulated degradation of specific tight junction proteins [23].

Notably, nutrient deprivation-induced autophagy has been shown to strengthen intestinal barrier integrity by promoting lysosomal-mediated degradation of the pore-forming tight junction protein claudin-2 [24], with recent studies elucidating the precise molecular mechanisms underlying this process. Conversely, dysregulated or excessive autophagic activity may precipitate detrimental consequences, including cellular metabolic stress, non-selective degradation of essential cellular components, and induction of autophagic cell death. This delicate balance underscores the complex, context-dependent nature of autophagy in intestinal pathophysiology, where its effects range from cytoprotective to potentially deleterious depending on the magnitude and duration of activation. CQ, a typical representative of the tetra-aminoquinoline class of compounds, is mainly used for malaria control in clinical practice. In terms of molecular mechanism, this drug can cause abnormal accumulation of LC3-II and inhibit the degradation of the p62 protein to exert autophagy inhibition [25].

Our mechanistic assays revealed that PSME2 knockdown increased LC3-II/LC3-I ratios, decreased SQSTM1/p62 levels, and enhanced LC3 puncta formation, indicative of autophagic flux activation. Importantly, pharmacological inhibition of autophagy using CQ abrogated the protective effects of PSME2 silencing, reinforcing the conclusion that PSME2 modulates inflammation and barrier integrity through autophagy. These findings provide proof-of-concept evidence for a PSME2–autophagy axis in UC pathogenesis. It should be emphasized that this study was specifically designed to investigate the role of PSME2 under inflammatory stress, which was modeled using LPS stimulation. Therefore, all knockdown experiments were performed under LPS challenge to mimic the inflamed epithelial environment relevant to UC pathophysiology. Thus, the essential control data for autophagy markers in the si-PSME2 setting are already included. We acknowledge, however, that si-PSME2 experiments were not performed under quiescent (non-LPS) conditions, and future studies will investigate whether PSME2 also regulates basal autophagy under homeostatic conditions.

Nevertheless, several caveats warrant consideration. First, CQ is a widely used autophagy inhibitor but exerts multiple off-target effects, including interference with endosomal trafficking and lysosomal acidification. Thus, while our results support a mechanistic link, they cannot be considered definitive. Future studies should employ more specific approaches, such as siRNA/shRNA targeting ATG5, ATG7, or BECN1, and real-time monitoring with mRFP-GFP-LC3 reporters to dissect autophagic flux dynamics more precisely.

Taken together, this study suggests that PSME2, functioning as a proteasome activator, may modulate autophagic flux in intestinal epithelial cells. This regulatory role appears to influence two key pathological features of UC: (1) impairment of intestinal barrier integrity and (2) dysregulated secretion of pro-inflammatory cytokines. The observed positive correlation between PSME2 expression levels and both histopathological severity and clinical disease activity supports its potential involvement in UC pathogenesis. These findings highlight PSME2 as a candidate of dual clinical relevance. These findings position PSME2 as both a potential biomarker and a candidate therapeutic target within the autophagy–inflammation axis. Future validation in advanced *in vivo* and human-derived models will be essential to establish its translational relevance and therapeutic potential.

## 5 Conclusions

This study suggests that PSME2 may contribute to UC pathology by impairing intestinal barrier integrity and promoting autophagy-associated inflammatory responses. Our results indicate that PSME2 upregulation correlates with disease severity in UC patients and experimental models. Mechanistically, PSME2 silencing was associated with improved epithelial barrier function and reduced inflammatory markers, while pharmacological autophagy inhibition partially reversed these effects. These findings provide preliminary evidence that PSME2 is involved in autophagy-related barrier dysfunction and inflammation in UC. Although the current data are insufficient to establish PSME2 as a diagnostic biomarker or therapeutic target, this study highlights it as a potential candidate warranting further *in vivo* validation and clinical investigation.

## References

[1] Ma Y, Yang D, Huang J, Liu K, Liu H, Wu H, et al. Probiotics for inflammatory bowel disease: is there sufficient evidence? Open Life Sci. 2024;19(1):20220821. 10.1515/biol-2022-0821 PMC10998680 38585636

[2] Shao J, Jin Y, Shao C, Fan H, Wang X, Yang G. Serum exosomal pregnancy zone protein as a promising biomarker in inflammatory bowel disease. Cell Mol Biol Lett. 2021;26(1):36. 10.1186/s11658-021-00280-x PMC8353742 34376139

[3] Li N, Zhao L, Geng X, Liu J, Zhang X, Hu Y, et al. Stimulation by exosomes from hypoxia-preconditioned hair follicle mesenchymal stem cells facilitates mitophagy by inhibiting the PI3K/AKT/mTOR signaling pathway to alleviate ulcerative colitis. Theranostics. 2024;14(11):4278–96. 10.7150/thno.96038 PMC11303078 39113800

[4] Rogler G, Singh A, Kavanaugh A, Rubin DT. Extraintestinal manifestations of inflammatory bowel disease: current concepts, treatment, and implications for disease management. Gastroenterology. 2021;161(4):1118–32. 10.1053/j.gastro.2021.07.042 PMC8564770 34358489

[5] Solitano V, Facciorusso A, Jess T, Ma C, Hassan C, Repici A, et al. Comparative risk of serious infections with biologic agents and oral small molecules in inflammatory bowel diseases: a systematic review and meta-analysis. Clin Gastroenterol Hepatol. 2023;21(4):907–21.e2. 10.1016/j.cgh.2022.07.032 35944832

[6] Li R, Yan L, Jiu J, Liu H, Li D, Li X, et al. PSME2 offers value as a biomarker of M1 macrophage infiltration in pan-cancer and inhibits osteosarcoma malignant phenotypes. Int J Biol Sci. 2024;20(4):1452–70. 10.7150/ijbs.90226 PMC10878157 38385075

[7] Rana PS, Ignatz-Hoover JJ, Guo C, Mosley AL, Malek E, Federov Y, et al. Immunoproteasome activation expands the MHC Class I immunopeptidome, unmasks neoantigens, and enhances t-cell anti-myeloma activity. Mol Cancer Ther. 2024;23(12):1743–60. 10.1158/1535-7163.MCT-23-0931 PMC11612626 39210605

[8] Wang X, Wu F, Deng Y, Chai J, Zhang Y, He G, et al. Increased expression of PSME2 is associated with clear cell renal cell carcinoma invasion by regulating BNIP3‑mediated autophagy. Int J Oncol. 2021;59(6):106. 10.3892/ijo.2021.5286 PMC8651225 34779489

[9] Kang Z, Wang J, Huang W, Liu J, Yan W. Identification of transcriptional heterogeneity and construction of a prognostic model for melanoma based on single-cell and bulk transcriptome analysis. Front Cell Dev Biol. 2022;10:874429. 10.3389/fcell.2022.874429 PMC9136400 35646893

[10] Qiu C, Wang W, Xu S, Li Y, Zhu J, Zhang Y, et al. Construction and validation of a hypoxia-related gene signature to predict the prognosis of breast cancer. BMC Cancer. 2024;24(1):402. 10.1186/s12885-024-12182-0 PMC10986118 38561760

[11] Wan C, Wu Q, Wang Y, Sun Y, Ji T, Gu Y, et al. Machine learning-based characterization of PANoptosis-related biomarkers and immune infiltration in ulcerative colitis: a comprehensive bioinformatics analysis and experimental validation. Int Immunopharmacol. 2025;151:114298. 10.1016/j.intimp.2025.114298 39986196

[12] Wu Y, Ran L, Yang Y, Gao X, Peng M, Liu S, et al. Deferasirox alleviates DSS-induced ulcerative colitis in mice by inhibiting ferroptosis and improving intestinal microbiota. Life Sci. 2023;314:121312. 10.1016/j.lfs.2022.121312 36563842

[13] Tang X, Wang W, Hong G, Duan C, Zhu S, Tian Y, et al. Gut microbiota-mediated lysophosphatidylcholine generation promotes colitis in intestinal epithelium-specific Fut2 deficiency. J Biomed Sci. 2021;28(1):20. 10.1186/s12929-021-00711-z PMC7958775 33722220

[14] Yang L, Wu G, Wu Q, Peng L, Yuan L. METTL3 overexpression aggravates LPS-induced cellular inflammation in mouse intestinal epithelial cells and DSS-induced IBD in mice. Cell Death Discov. 2022;8(1):62. 10.1038/s41420-022-00849-1 PMC8844074 35165276

[15] Khan S, Mentrup HL, Novak EA, Siow VS, Wang Q, Crawford EC, et al. Cyclic GMP-AMP synthase contributes to epithelial homeostasis in intestinal inflammation via Beclin-1-mediated autophagy. Faseb J. 2022;36(5):e22282. 10.1096/fj.202200138R PMC9040047 35344224

[16] Ganapathy AS, Saha K, Suchanec E, Singh V, Verma A, Yochum G, et al. AP2M1 mediates autophagy-induced CLDN2 (claudin 2) degradation through endocytosis and interaction with LC3 and reduces intestinal epithelial tight junction permeability. Autophagy. 2022;18(9):2086–103. 10.1080/15548627.2021.2016233 PMC9466623 34964704

[17] Kempuraj D, Mohan RR. Autophagy in extracellular matrix and wound healing modulation in the cornea. Biomedicines. 2022;10(2):339. 10.3390/biomedicines10020339 PMC8961790 35203548

[18] Sahoo DK, Heilmann RM, Paital B, Patel A, Yadav VK, Wong D, et al. Oxidative stress, hormones, and effects of natural antioxidants on intestinal inflammation in inflammatory bowel disease. Front Endocrinol (Lausanne). 2023;14:1217165. 10.3389/fendo.2023.1217165 PMC10493311 37701897

[19] Sina C, Kemper C, Derer S. The intestinal complement system in inflammatory bowel disease: shaping intestinal barrier function. Semin Immunol. 2018;37:66–73. 10.1016/j.smim.2018.02.008 29486961

[20] Sun K, Liu YX, Li MX, Qi CH, Wu XS, Kang YA, et al. PSME2 promotes malignant progression through autophagy modulation via IL-6/STAT3 signaling pathway in esophageal squamous cell carcinoma. Life Sci. 2025;376:123749. 10.1016/j.lfs.2025.123749 40404117

[21] Zhang X, Wei M, Fan J, Yan W, Zha X, Song H, et al. Ischemia-induced upregulation of autophagy preludes dysfunctional lysosomal storage and associated synaptic impairments in neurons. Autophagy. 2021;17(6):1519–42. 10.1080/15548627.2020.1840796 PMC8205014 33111641

[22] Ma Z, Wu J, Wu Y, Sun X, Rao Z, Sun N, et al. Parkin increases the risk of colitis by downregulation of VDR via autophagy-lysosome degradation. Int J Biol Sci. 2023;19(5):1633–44. 10.7150/ijbs.77153 PMC10086757 37056928

[23] Zhang H, Cui Z, Cheng D, Du Y, Guo X, Gao R, et al. RNF186 regulates EFNB1 (ephrin B1)-EPHB2-induced autophagy in the colonic epithelial cells for the maintenance of intestinal homeostasis. Autophagy. 2021;17(10):3030–47. 10.1080/15548627.2020.1851496 PMC8525924 33280498

[24] Ahmad R, Kumar B, Tamang RL, Talmon GA, Dhawan P, Singh AB. P62/SQSTM1 binds with claudin-2 to target for selective autophagy in stressed intestinal epithelium. Commun Biol. 2023;6(1):740. 10.1038/s42003-023-05116-2 PMC10352296 37460613

[25] Cocco S, Leone A, Roca MS, Lombardi R, Piezzo M, Caputo R, et al. Inhibition of autophagy by chloroquine prevents resistance to PI3K/AKT inhibitors and potentiates their antitumor effect in combination with paclitaxel in triple negative breast cancer models. J Transl Med. 2022;20(1):290. 10.1186/s12967-022-03462-z PMC9235112 35761360

